# Gray Matter Network Associated With Attention in Children With Attention Deficit Hyperactivity Disorder

**DOI:** 10.3389/fpsyt.2022.922720

**Published:** 2022-07-04

**Authors:** Xing-Ke Wang, Xiu-Qin Wang, Xue Yang, Li-Xia Yuan

**Affiliations:** ^1^Jing Hengyi School of Education, Hangzhou Normal University, Hangzhou, China; ^2^Center for Cognition and Brain Disorders, The Affiliated Hospital of Hangzhou Normal University, Hangzhou, China; ^3^TMS Center, Deqing Hospital of Hangzhou Normal University, Zhejiang, China; ^4^Institute of Psychological Sciences, Hangzhou Normal University, Hangzhou, China; ^5^Zhejiang Key Laboratory for Research in Assessment of Cognitive Impairments, Hangzhou Normal University, Hangzhou, China

**Keywords:** attention deficit hyperactivity disorder (ADHD), structural MRI (sMRI), gray matter volume, scaled subprofile model of principal component analysis (SSM-PCA), ADHD-200, gray matter network, inattention

## Abstract

**Background:**

Attention deficit hyperactivity disorder (ADHD) is one of the most prevalent childhood-onset neurodevelopmental disorders; however, the underlying neural mechanisms for the inattention symptom remain elusive for children with ADHD. At present, the majority of studies have analyzed the structural MRI (sMRI) with the univariate method, which fails to demonstrate the interregional covarying relationship of gray matter (GM) volumes among brain regions. The scaled subprofile model of principal component analysis (SSM-PCA) is a multivariate method, which can detect more robust brain-behavioral phenotype association compared to the univariate analysis method. This study aims to identify the GM network associated with attention in children with ADHD by applying SSM-PCA to the sMRI.

**Methods:**

The sMRI of 209 children with ADHD and 209 typically developing controls (TDCs) aged 7–14 years from the ADHD-200 dataset was used for anatomical computation, and the GM volume in each brain region was acquired. Then, SSM-PCA was applied to the GM volumes of all the subjects to capture the GM network of children with ADHD (i.e., ADHD-related pattern). The relationship between the expression of ADHD-related pattern and inattention symptom was further investigated. Finally, the influence of sample size on the analysis of this study was explored.

**Results:**

The ADHD-related pattern mainly included putamen, pallium, caudate, thalamus, right accumbens, superior/middle/inferior frontal cortex, superior occipital cortex, superior parietal cortex, and left middle occipital cortex. In addition, the expression of the ADHD-related pattern was related to inattention scores measured by the Conners’ Parent Rating Scale long version (CPRS-LV; *r* = 0.25, *p* = 0.0004) and the DuPaul ADHD Rating Scale IV (ADHD-RS; *r* = 0.18, *p* = 0.03). Finally, we found that when the sample size was 252, the results of ADHD-related pattern were relatively reliable. Similarly, the sample size needed to be 162 when exploring the relationship between ADHD-related pattern and behavioral indicator measured by CPRS-LV.

**Conclusion:**

We captured a GM network associated with attention in children with ADHD, which is different from that in adolescents and adults with ADHD. Our findings may shed light on the diverse neural mechanisms of inattention and provide treatment targets for children with ADHD.

## Introduction

Attention deficit hyperactivity disorder (ADHD) is one of the most prevalent childhood-onset neurodevelopmental disorders characterized by inattention and/or hyperactivity and impulsivity, which impaired the school functioning and academic achievement of schoolchildren (1). The worldwide prevalence rate of children with ADHD is 7.2% (2), which arouses more and more concerns from researchers. Although ADHD is a highly heterogeneous disorder with three clinical subtypes, inattention is one of the core symptoms and rarely vanishes compared to the significant relief of hyperactive/impulsive symptoms over time (3–5). However, the underlying neural mechanisms for the inattention symptom of children with ADHD remain elusive.

For children with ADHD, the anatomical abnormalities in the brain volume have been widely investigated through structural magnetic resonance imaging (sMRI), shedding light on the disease’s mechanisms and treatments. In a recent meta-analysis of gray matter (GM), children with ADHD showed a decreased GM volume in the right globus pallidus, putamen, and bilateral caudate (6). Moreover, based on the ENIGMA-ADHD sample, the volumes of accumbens, amygdala, caudate, hippocampus, and putamen were found to be smaller in children with ADHD (7). So far, the majority of previous sMRI studies have applied univariate analysis methods, which provide local information but fail to demonstrate the interregional covarying relationship of GM volumes among different brain regions (8). However, the human brain is a complicated network, in which different brain regions interact with each other functionally and structurally (9). Moreover, recognizing the importance of understanding the function, organization, and development of interacting brain regions for the past few years, recent neuroimaging research has shifted its focus from discrete neural substrates to the role of distributed neural networks (10). Focusing on abnormalities of ADHD-related neural networks rather than discrete neural substrates coincides with the development trend of the neuroimaging research. For adults and adolescents, GM network identified in adults compromising the bilateral cerebellar tonsil and culmen and GM network identified in adolescents compromising the left cerebellar region were significantly associated with inattention (11). Yet, few sMRI studies have investigated the brain network changes based on GM in children with ADHD, which may uncover the neural mechanism of ADHD and provide an objective basis for the diagnosis of ADHD.

The scaled subprofile model of principal component analysis (SSM-PCA) is a multivariate method for network analysis (12), which can detect more robust brain-behavioral phenotype association compared to univariate analysis methods (13). SSM-PCA views various brain regions as interrelated and coordinated nodes of an integrated network (8) and can reveal the brain pattern characteristics of specific diseases based on sMRI (14–16). Since SSM-PCA can capture the subtle changes caused by the disease (8), it may be helpful to capture the abnormal structural brain network in ADHD. To date, SSM-PCA had been widely applied to explore the influence of many neurological and psychiatric illnesses on sMRI, such as Parkinson’s disease (8), Alzheimer’s disease (17), and Spinocerebellar ataxia type 3 (18). However, SSM-PCA has not been used for analyzing the GM in children with ADHD, yet.

Thus, this study aimed to identify the GM network of children with ADHD (i.e., ADHD-related pattern) by applying the SSM-PCA approach to sMRI and investigating the relationship between the ADHD-related pattern and inattention symptom. According to previous studies, we hypothesized that the ADHD-related pattern would comprise brain regions in frontal cortex, basal ganglia, and cerebellum, and the expression of ADHD-related pattern would relate to inattention scores.

## Materials and Methods

### Participants and Data Acquisition

The data used in this study are publicly obtained from the ADHD-200 Consortium, which can be downloaded from the website of NeuroImaging Tools & Resources Collaboratory Image Repository.^1^ The ADHD-200 cohort contains 776 subjects with anatomical images aggregated from 8 independent sites (age range: 7–21 years). Demographic data including age, sex, handedness, secondary diagnosis, and medication status are also available from the website. The diagnosis of ADHD is based on the Conners’ Parent Rating Scale long version (CPRS-LV) or DuPaul ADHD Rating Scale IV (ADHD-RS) and is divided into three subtypes, including inattentive type (ADHD-I), hyperactive/impulsive type (ADHD-H), and combined type (ADHD-C). For children measured with CPRS-LV, the diagnosis of ADHD was based on a T-score greater than or equal to 65 on at least one ADHD-related index (19). For children measured with ADHD-RS, the diagnosis of ADHD needed to meet the criterion, namely, six of nine items scored 2 or 3 from at least one ADHD-related item (20). According to a previous study, we defined the age range of children as 7–14 years (7).

**FIGURE 1:**
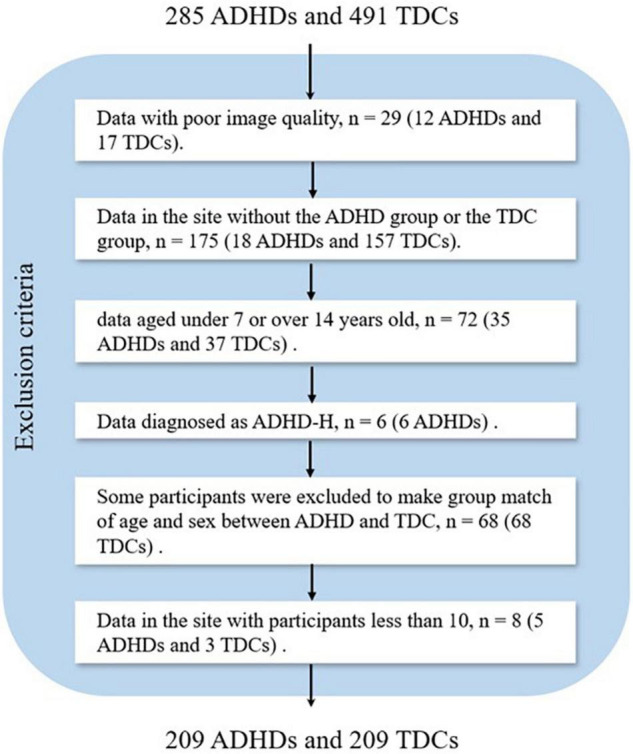
Flowchart of data exclusion for ADHD-200 (ADHD, attention deficit hyperactivity disorder; TDC, typically developing control subject).

In this study, we only analyzed the T1-weighted sMRI dataset. The exclusion criteria for the data were as follows: (1) data with poor image quality; (2) data in the site without the ADHD group or the typically developing control (TDC) group; (3) data aged under 7 years or over 14 years; (4) data diagnosed as ADHD-H; (5) some data were excluded to make group match of age and sex between ADHD and TDC; and (6) data in the site with participants less than 10. In total, 358 subjects (76 ADHDs and 282 TDCs) were excluded, and the number of the excluded subjects in each step is presented in Figure 1. Finally, 418 participants were included, and the demographic information of all the participants was presented in Table 1.

**TABLE 1:** Demographic information of all the participants (ADHD, attention deficit hyperactivity disorder; TDC, typically developing control subject; C, ADHD-Combined; I, ADHD-Inattentive; CPRS-LV, Conners’ Parent Rating Scale long version; ADHD-RS, DuPaul ADHD Rating Scale IV).

	ADHD (*N* = 209)	TDC (*N* = 209)	Statistics
Male/Female (*n*)	162/47	150/59	χ^2^ = 1.82; *p* = 0.18
Age (mean ± SD years)	10.81 ± 2.11	10.84 ± 1.90	*t*_(416)_ = -0.17; *p* = 0.87
Subtype (C/I)	130/79	–	–
Subject number measured by CPRS-LV/ADHD-RS	108/66	90/76	χ^2^ = 2.15; *p* = 0.14
Inattention scores of CPRS-LV (mean ± SD)	71.42 ± 9.81	45.62 ± 5.34	*t*_(196)_ = 23.46; *p* < 0.001
Inattention scores of ADHD-RS (mean ± SD)	28.48 ± 3.47	15.33 ± 3.66	*t*_(140)_ = 21.95; *p* < 0.001

### Gray Matter Volume Calculation

Computational Anatomy Toolbox 12 (CAT12.7 r1739^2^) developed by Christian Gaser and Robert Dahnke (Jena University Hospital, Departments of Psychiatry and Neurology) is an extension of Statistical Parametric Mapping 12 (SPM12^3^) for anatomical computation. The segmentation and normalization were implemented using DARTEL, which utilized a single constant velocity field to generate diffeomorphic and invertible deformations. The GM segments of each participant were non-linearly registered to the automated anatomical labeling 3 (AAL3) (21) template to get the GM volume of each region. The actual GM volume measured in cubic centimeters is referred to as the absolute volume. A ratio of the absolute volume to the total intracranial volume is used for further analysis (22).

### Multisite Effect Correction

Since the ADHD-200 cohort consists of datasets from different institutions, imaging data suffer from site, collection time, and data acquisition parameter variability across data collections. The site effect was removed using the ComBat function available in MATLAB^4^ (23, 24). The ComBat is a popular batch-effect correction tool used in genomics (25), which also performs well for multisite effect correction for neuroimaging (23, 24). During Combat, the diagnosis, age, and sex were treated as interested biological variables, and a default non-parametric prior method was used in the empirical Bayes procedure.

### Statistical Analysis

#### Scaled Subprofile Model of Principal Component Analysis on Gray Matter Volume

An in-house developed SSM-PCA toolbox was used for performing SSM-PCA on GM volume from the ADHD-200 dataset (26). As the GM volume varied a lot among brain regions, it was normalized by z-transformation among subjects before SSM-PCA. The general process of SSM-PCA is as follows: (1) the GM volume of the ADHD and the TDC groups was placed together in an *M* × *N* dimensional data matrix, where M is the number of brain regions and N is the number of the sum of ADHD and TDC participants, and each column represented the GM volume of all the brain regions of a participant; (2) each column was centered to zero by subtracting the mean of each column; (3) each row was centered to zero by subtracting the mean of each row, and then the subject residual profile (SRP) was obtained; (4) the reduced singular value decomposition (SVD) was utilized to factorize the SRP:


(1) $$U\Sigma V^{T}=SVD\left(SRP\right)$$

where *U* is an *M* × *N* matrix composed of the left unit-normalized orthogonal singular vectors as columns, *****Σ***** is an *N* × *N* diagonal matrix composed of singular values *σ_*k*_*, where *k* is the component number, and *V* is an *N* × *N* matrix composed of the right unit-normalized orthogonal singular vectors as columns; and (5) the group invariant subprofile (GIS; i.e., patterns) and subject scaling factor (SSF; i.e., patterns’ expressions in each subject) can be computed by:


(2) $$GIS_{ik}=U_{ik}$$

(3) $$SSF_{jk}=\sum_{i=1}^{M}\left(SRP_{ij}\times GIS_{ik}\right)$$

where *i* is the region number and *j* is the subject number (6). The ratio of variance corresponding to each GIS to the total variance was calculated and named the variance accounting for (VAF).

Each value in the GIS matrix indicates a weight representing the contribution of a region to the corresponding pattern. Regions with relatively large weights in the pattern constitute the “network” called in some papers (12, 26).

#### Gray Matter Network: Attention Deficit Hyperactivity Disorder-Related Pattern

According to the elbow point of the VAF curve of all the patterns, the first 10 patterns were used for further analysis. Then, the difference between the ADHD group and TDC group was assessed by performing the two-sample *t*-test on the first 10 SSFs with controlling for age and sex and found that the first, sixth, and tenth SSFs showed a significant difference between the two groups (*p* < 0.05). Finally, an ADHD-related pattern was identified by a linear combination of the first, sixth, and tenth patterns. According to previous research (27), the linear combination coefficient was calculated by conducting a logistic regression analysis on the SSFs of the first, sixth, and tenth patterns to maximize the difference between the two groups. The sign of the ADHD-related pattern was defined such that patients with ADHD had elevated mean expression relative to the controls.

#### Relationship Between Attention Deficit Hyperactivity Disorder-Related Pattern and Behavioral Indicator

As the datasets come from different scanning sites and the behavior scales vary among sites, the sample was divided into three subsamples, i.e., 198 participants measured by CPRS-LV, 142 participants measured by ADHD-RS, and 78 participants without inattention scores. Then, the Spearman correlation was performed to investigate the relationship between inattention scores and the expression of ADHD-related pattern on the two subsamples measured by CPRS-LV and ADHD-RS, separately. The above statistical analysis was based on Statistical Product and Service Solutions (SPSS 21.0^5^).

#### Sample Size Effect on the Analysis

As studies with a small sample size are vulnerable to sampling variability (13), here, we investigated the influence of sample size on the results of this study. Since participants consisted of three subsamples, stratified sampling was used according to the population ratio of three subsample datasets, namely, 3:2:1 (CPRS-LV: ADHD-RS: participants without the inattention scores).

The detailed steps to investigate the sample size effect on the analysis were as follows: (1) first, a certain number of participants were randomly selected from the ADHD group and the TDC group to form a new sample. Due to the limitation of the number of subjects in each group, the maximum sample size was 198. Here, the sample size of each group was set as 54:18:198, and 18 subjects consisted of 9 subjects measured by CPRS-LV, 6 subjects measured by ADHD-RS, and 3 subjects without the inattention scores. (2) The SSFs of the ADHD-related pattern of the selected subjects were used to perform the group difference and correlation analysis with inattention scores from CPRS-LV and ADHD-RS. Steps 1 and 2 were repeated 1,000 times.

## Results

### Gray Matter Network: Attention Deficit Hyperactivity Disorder-Related Pattern

**FIGURE 2:**
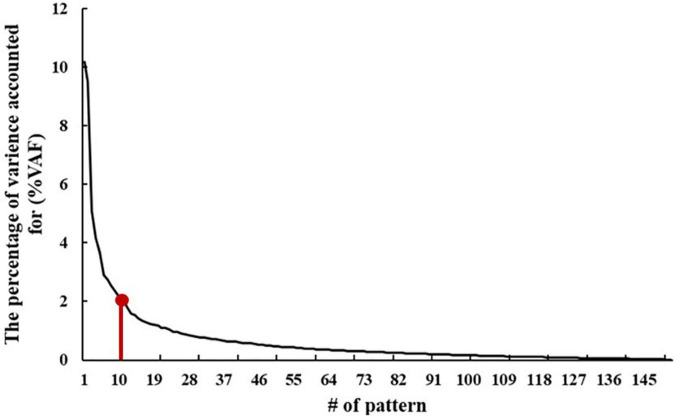
The percentage of the variance accounting for (%VAF) for each pattern.

**FIGURE 3:**
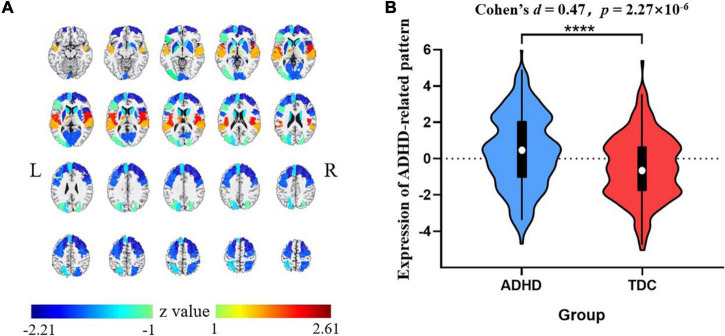
The z-transformed ADHD-related pattern (with |z| > 1) **(A)** and its expression distribution in the ADHD and TDC groups (the slices displayed from −15 mm to +80 mm at axis view) **(B)**. Positive and negative *z*-values represented increased and reduced GM volumes in the ADHD group than TDC group, respectively. ****p < 0.0001. ADHD, attention deficit hyperactivity disorder; TDC, typically developing control subject.

The VAF of each pattern is shown in Figure 2, and the elbow point of the VAF curve was 10, so the first 10 patterns were used for further analysis. The first, sixth, and tenth SSFs of the first 10 patterns showed a significant difference between the ADHD group and TDC group (*p* < 0.05) (Table 2). After a linear combination of these three patterns, the ADHD-related pattern significantly discriminates the ADHD and TDC groups [*t*_(416)_ = 4.80, Cohen’s *d* = 0.47, 95% CI = (0.27, 0.66); *p* = 2.27 × 10^–6^] (Figure 3B). The topography of z-transformed ADHD-related pattern with a threshold of |z| > 1 is shown in Figure 3A. Positive z-value represented an increased GM volume in the ADHD group than in the TDC group, mainly including the bilateral thalamus, superior temporal cortex, and cerebellum crus I. Inversely, a negative z-value represented a decreased GM volume in the ADHD group than in the TDC group, mainly involving putamen, pallium, caudate, superior/middle/inferior frontal cortex, superior occipital cortex, superior parietal cortex, right accumbens, and left middle occipital cortex.

**TABLE 2:** The two-sample *t*-test results of SSFs of the first 10 patterns between ADHD and TDC group.

	SSF1	SSF2	SSF3	SSF4	SSF5	SSF6	SSF7	SSF8	SSF9	SSF10
*p*-value	0.01	0.79	0.75	0.51	0.44	0.0008	0.91	0.43	0.37	0.04
*T*-value	2.58	0.27	0.33	0.65	–0.77	–3.39	0.12	–0.78	–0.89	–2.09
Cohen’s *d*	0.25	0.02	0.03	0.06	–0.08	–0.33	0.01	–0.08	–0.09	–0.20

*For example, the p-value of 0.01 meant the t-test result for the expression of the first pattern (i.e., SSF1) between the ADHD and TDC groups (ADHD, attention deficit hyperactivity disorder; TDC, typically developing control subject; SSF, subject scaling factor).*

### Relationship Between Attention Deficit Hyperactivity Disorder-Related Pattern and Inattention Scores

**FIGURE 4:**
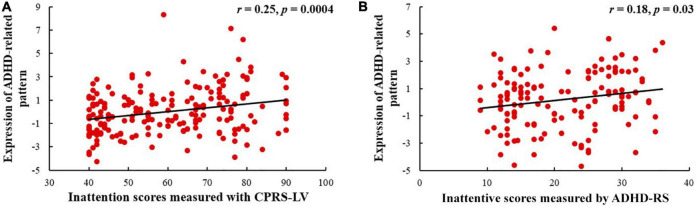
Relationships between the expression of ADHD-related pattern and inattentive scores measured by CPRS-LV **(A)** and ADHD-RS **(B)** (ADHD, attention deficit hyperactivity disorder; CPRS-LV, Conners’ Parent Rating Scale long version; ADHD-RS, DuPaul ADHD Rating Scale IV).

As shown in Figure 4, the expression of the ADHD-related pattern was positively related to inattention scores of CPRS-LV (*n* = 198, *r* = 0.25, *p* = 0.0004). Similarly, the expression of the ADHD-related pattern was significantly associated with inattention scores of ADHD-RS (*n* = 142, *r* = 0.18, *p* = 0.031).

### Sample Size Effect on the Analysis

**FIGURE 5:**
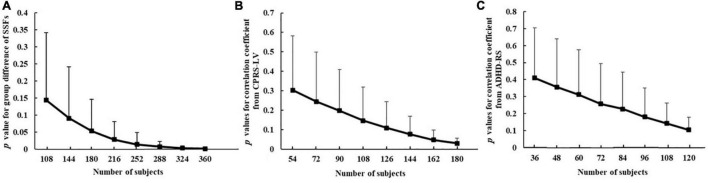
The relationship between sample size and *p*-value for group difference of SSFs of the ADHD-related pattern between ADHD group and TDC group **(A)**, correlation coefficient between ADHD-related pattern’s expression and inattention scores from CPRS-LV **(B)**, and ADHD-RS **(C)** (ADHD, attention deficit hyperactivity disorder; TDC, typically developing control subject; CPRS-LV, Conners’ Parent Rating Scale long version; ADHD-RS, DuPaul ADHD Rating Scale IV).

Figure 5 shows the changing tendency of *p*-values with the sample size. The difference in SSF of the ADHD-related pattern between ADHD group and TDC group became more and more significant with the expansion of the sample size. The expression of the pattern showed significant differences between the two groups when the sample size was 180. In addition, when the sample size was 252, the *p*-value for SSF reached a plateau and its range became smaller and smaller. Similarly, the expression of the pattern was significantly associated with inattention scores measured by CPRS-LV when the sample size was 162. Due to the small sample size of subjects measured by ADHD-RS, the sample size effect of ADHD-RS remained elusive. In general, these results showed that sampling variability decreased and the associations stabilized along with increasing sample sizes.

## Discussion

In this study, we performed SSM-PCA on GM volume from ADHD-200 datasets and captured the ADHD-related pattern, which mainly included the putamen, pallium, caudate, thalamus, right accumbens, superior/middle/inferior frontal cortex, superior occipital cortex, superior parietal cortex, and left middle occipital cortex. In addition, the expression of the ADHD-related pattern was significantly associated with inattention scores.

### Gray Matter Network: Attention Deficit Hyperactivity Disorder-Related Pattern

As shown in the ADHD-related pattern, our results highlight the importance of basal ganglia (caudate, putamen, and accumbens) and bilateral superior/middle/inferior frontal cortex in ADHD. These regions demonstrated a significant reduction of GM volume in ADHD relative to TDC. These findings are in line with our hypotheses and similar brain areas and GM volume changes have been reported in previous studies (6, 7, 28–33). Moreover, the basal ganglia are a key component of the dopaminergic mesolimbic system (34, 35) and play a crucial role in goal-directed behaviors, motivation and reward processing, and motor control, all of which are known to be deficient in ADHD (10, 36, 37). The frontal cortex has been shown to play a key role in cognitive processes, which are related to the impairment of maintaining and shifting attention in ADHD (38, 39).

This study finds increased GM volumes in the thalamus, superior temporal cortex, and cerebellum lobule I. For the thalamus, our result is consistent with a previous study in patients with ADHD-I from the ADHD-200 datasets (40). Furthermore, the thalamus is likely associated with arousal and motivation (41) and the underarousal and sleep disturbance are common co-occurring symptoms of ADHD (42). Therefore, the thalamus should be taken seriously in the treatment of ADHD. Reduced cerebellum (i.e., cerebellum lobule V) and middle temporal cortex volume have been reported in children with ADHD (43–47); however, our results illustrated increased GM in cerebellum crus I and superior temporal cortex. Such inconsistency may result from the subject heterogeneity, as differences between different ADHD subtypes have been demonstrated (48–50). Contrary to previous studies, which included all the subtypes, we excluded the ADHD-H subtype. In addition, the age difference between used subjects may also lead to such inconsistency. Duan et al. investigated GM networks of two cohorts (adults and adolescents) and found that adolescent patients showed different GM volume changes than that of adult patients (11).

### Relationship Between Attention Deficit Hyperactivity Disorder-Related Pattern and Inattention Scores

In our study, the expressions of ADHD-related pattern were significantly associated with inattention scores measured with two different scales (i.e., CPRS-LV and ADHD-RS). Our findings are consistent with the previous studies (11, 51). Castellanos et al. showed that GM volumes of caudate, frontal cortex, temporal cortex, and cerebellum were correlated with the score of attention problems in children with ADHD (51). The brain regions mentioned earlier were also captured in the ADHD-related pattern here. For adults and adolescents, GM network compromising the cerebellar tonsil and culmen identified in adults and GM network compromising the left cerebellar region identified in adolescents were significantly associated with inattention (11). In this study, we also found that GM network compromising cerebellar regions was associated with inattention scores in children with ADHD. Different from adults and adolescents, our results illustrated increased GM volumes in cerebellum crus I and highlighted the reduced GM volumes in basal ganglia and superior/middle/inferior frontal cortex in children with ADHD.

### Comparison Between Attention-Related Gray Matter Networks in Children, Adolescents, and Adults With Attention Deficit Hyperactivity Disorder

In adolescents with ADHD, previous research identified an attention-related GM network compromising the bilateral cerebellar tonsil and culmen (11). In adults with ADHD, the attention-related GM network consists of the left cerebellar region (11). In contrast, in children with ADHD, the attention-related GM network mainly includes the putamen, pallium, caudate, thalamus, right accumbens, superior/middle/inferior frontal cortex, superior occipital cortex, superior parietal cortex, and left middle occipital cortex. The abovementioned evidence illustrates that the brain regions involved in attention evolve with age, which indicates that different neural mechanisms of attention deficit disorder are possible and inattention may not be a single syndrome (52). Clinically, different treatment options exist and the practical experience is the impossibility of only one medical treatment (52). Collectively, the current diagnosis of ADHD is still based on behavior performance, which may be the result of diverse causes and etiologies. Thus, the development of effective biological test is emergent for its diagnosis and treatment.

### Sample Size Effect on the Analysis

This study not only focused on the ADHD-related pattern but also investigated the effect of sample size on the whole data analysis. Our results showed that the difference between the ADHD group and the TDC group was enhanced with the expansion of sample size and reached stable with a sample size greater than 252. Similarly, when exploring the relationship between ADHD-related pattern and behavioral indicator measured by CPRS-LV, the sample size needs to be larger than 162. The required sample size is much less than that needed in univariate analysis methods (13). These results demonstrated that SSM-PCA can detect robust brain-behavioral phenotype association.

## Limitations

This study also has some limitations. First, the inattention scores of participants from different sites in the ADHD-200 dataset were measured with different scales (i.e., CPRS-LV and ADHD-RS), which forces us to investigate the correlation between inattention scores and ADHD-related pattern expression, separately. Second, to test the robustness of our results, replication on other datasets is necessary. Third, due to the small number of subjects in the subtypes of ADHD-I and ADHD-C, we analyzed all the data together; however, it is important to further investigate the subject heterogeneity between these two ADHD subtypes regarding attention-related GM pattern, separately.

## Conclusion

We obtained a GM network associated with attention in children with ADHD, which is different from that in adolescents and adults with ADHD. Our findings may shed light on the diverse neural mechanisms of inattention and provide treatment targets for children with ADHD.

## Data Availability Statement

The original contributions presented in this study are included in the article/supplementary material, further inquiries can be directed to the corresponding author.

## Ethics Statement

The studies involving human participants were reviewed and approved by the NITRC and 1000 Functional Connectomes Project. Written informed consent to participate in this study was provided by the participants’ legal guardian/next of kin. Written informed consent was obtained from the individual(s), and minor(s)’ legal guardian/next of kin, for the publication of any potentially identifiable images or data included in this article.

## Author Contributions

L-XY conceived and designed the experiment. X-KW performed the data analysis. X-QW and XY provided advice on the analysis and interpretation of the results. X-KW and L-XY wrote the manuscript. All authors contributed to the article and approved the submitted version.

## Conflict of Interest

The authors declare that the research was conducted in the absence of any commercial or financial relationships that could be construed as a potential conflict of interest.

## Publisher’s Note

All claims expressed in this article are solely those of the authors and do not necessarily represent those of their affiliated organizations, or those of the publisher, the editors and the reviewers. Any product that may be evaluated in this article, or claim that may be made by its manufacturer, is not guaranteed or endorsed by the publisher.

## Footnotes

1
https://www.nitrc.org/ir/

2
http://www.neuro.uni-jena.de/cat/

3
http://www.fil.ion.ucl.ac.uk/spm

4
https://github.com/Jfortin1/ComBatHarmonization

5
https://www.ibm.com/cn-zh/analytics/spss-statistics-software
